# Electrochemical Lithium Storage Performance of Molten Salt Derived V_2_SnC MAX Phase

**DOI:** 10.1007/s40820-021-00684-6

**Published:** 2021-07-22

**Authors:** Youbing Li, Guoliang Ma, Hui Shao, Peng Xiao, Jun Lu, Jin Xu, Jinrong Hou, Ke Chen, Xiao Zhang, Mian Li, Per O. Å. Persson, Lars Hultman, Per Eklund, Shiyu Du, Zhifang Chai, Zhengren Huang, Na Jin, Jiwei Ma, Ying Liu, Zifeng Lin, Qing Huang

**Affiliations:** 1 Engineering Laboratory of Advanced Energy Materials, Ningbo Institute of Industrial Technology, Chinese Academy of Sciences, Ningbo, 315201 Zhejiang People’s Republic of China; 2 Qianwan Institute of CNiTECH, Ningbo, 315336 People’s Republic of China; 3 College of Materials Science and Engineering, Sichuan University, Chengdu, 610065 People’s Republic of China; 4 CIRIMAT UMR CNRS 5085, Université Toulouse III- Paul Sabatier, 118 route de Narbonne, 31062 Toulouse Cedex 9, France; 5 Department of Physics, Chemistry and Biology (IFM), Linköping University, 58183 Linköping, Sweden; 6 School of Machine Engineering, Dongguan University of Technology, Dongguan, 523808 People’s Republic of China; 7 Institute of New Energy for Vehicles, School of Materials Science and Engineering, Tongji University, Shanghai, 201804 People’s Republic of China

**Keywords:** MAX phase, Molten salt, Lithium storage, High-rate, Energy storage

## Abstract

MAX phases are gaining attention as precursors of two-dimensional MXenes that are intensively pursued in applications for electrochemical energy storage. Here, we report the preparation of V_2_SnC MAX phase by the molten salt method. V_2_SnC is investigated as a lithium storage anode, showing a high gravimetric capacity of 490 mAh g^−1^ and volumetric capacity of 570 mAh cm^−3^ as well as superior rate performance of 95 mAh g^−1^ (110 mAh cm^−3^) at 50 C, surpassing the ever-reported performance of MAX phase anodes. Supported by *operando* X-ray diffraction and density functional theory, a charge storage mechanism with dual redox reaction is proposed with a Sn–Li (de)alloying reaction that occurs at the edge sites of V_2_SnC particles where Sn atoms are exposed to the electrolyte followed by a redox reaction that occurs at V_2_C layers with Li. This study offers promise of using MAX phases with M-site and A-site elements that are redox active as high-rate lithium storage materials.

**Supplementary Information:**

The online version contains supplementary material available at 10.1007/s40820-021-00684-6.

## Highlights

- Small size V_2_SnC MAX phase was prepared by the molten salt method.
- V_2_SnC MAX phase electrode is able to deliver high gravimetric capacity up to 490 mAh g^−1^ and volumetric capacity of 570 mAh cm^−3^
- A charge storage mechanism with V_2_C-Li redox and Sn–Li alloying dual reactions was proposed

**Supplementary Information:**

The online version contains supplementary material available at 10.1007/s40820-021-00684-6.

## Graphical abstract

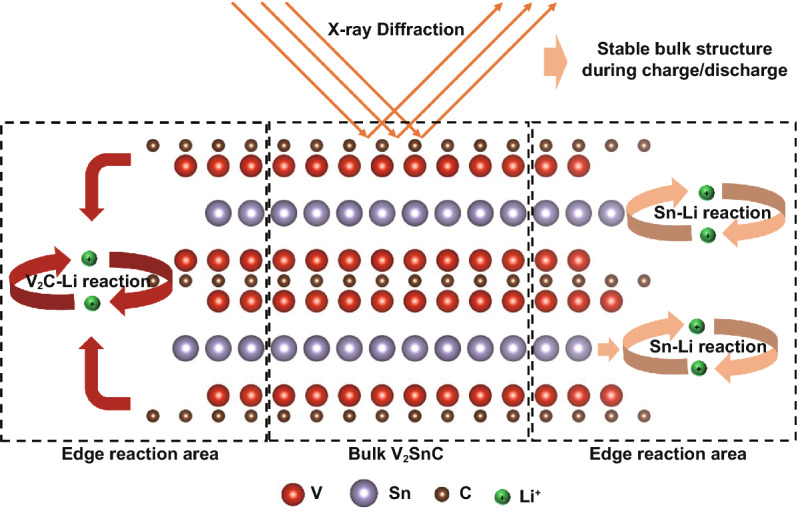

## Introduction

The MAX phases are hexagonal ternary metal carbides and/or nitrides with a general formula M_*n*+1_AX_*n*_, where M is a transition metal, A is primarily a group 13–16 element, X is carbon and/or nitrogen, and *n* is typically 1, 2, or 3 [1]. MAX phases (including solid solutions and ordered quaternary phases) were synthesized by common synthesis methods such as hot pressing (HP) and spark plasma sintering (SPS) [2]. Compared to HP and SPS, molten salt method (MSM) is a simple and cost-effective route for preparing MAX phase powders, which has attracted broaden appeal in recent years. Molten salt synthesis is a modification of the powder metallurgical method by adding low melting point salts to the reactants for synthesizing at temperature above the melting point of the salts, where molten salts were used as the solvents and/or reacting species [3]. As a high-temperature ionic solvent, the molten salt bath offers high solvation power and liquid environment for reactants that facilitate the mass transport and nucleation processes. The synthesis temperature and time could be reduced to obtain more fine and uniform particles [4]. Some MAX phases (e.g., Cr_2_AlC, Ti_3_SiC_2_, V_2_AlC, Ti_3_AlC_2_, Ti_2_AlN) have been synthesized by molten salt method with small size at relatively low temperatures [3, 5–8].

Lithium-ion batteries (LIBs) as one of the key electrochemical energy storage power sources have witnessed tremendous progress with many energy storage materials are developed [9–11]. Considering the laminated structure, metallic conductivity, MAX phases are potentially excellent lithium storage hosts. Xu et al. [12] reported on the reversible electrochemical intercalation of Li^+^ ions into Ti_2_SC and Ti_3_SiC_2_ MAX phases. By reducing the particle size, the Ti_2_SC MAX phase delivered an initial capacity of 80 mAh g^–1^ (0.4 A g^–1^), increasing to 180 mAh g^–1^ after 1000 cycles. Chen et al*.* [13] demonstrated that the partially etched Ti_3_AlC_2_ MAX phase is found to have a much higher capacity (160 mAh g^−1^ at 1C) than its corresponding Ti_3_C_2_T_*x*_-MXene thanks to the extra capacity originating from the formation of Li-Al alloy. An oxygen doped Ti_3_SiC_2_ MAX was also reported with an initial capacity of 70 mAh g^−1^ at 1 A g^−1^ and subsequent increase to 180 mAh g^−1^ after 3000 cycles [14]. Metallic Sn, as one promising alloy anode, had attracted broaden appeal due to its high theoretical capacity [15–17]. Zhao et al*.* [18] reported the Sn–MAX phase (Nb_2_SnC) with a capacity of 234 mAh g^−1^ at 0.05 A g^−1^ and identified the additional capacity contribution as the Sn–Li alloying reaction. Therefore, these few works dedicated to investigating MAX phases as lithium storage hosts attest that MAX phases (especially Sn-MAX) show promise as electrode materials for Li-ion batteries (LIBs).

In 2020, a new V_2_SnC MAX phase was first reported by Xu et al. [19] via solid phase reaction of V, Sn, and C mixtures. Thus, further investigation on synthesizing this new V_2_SnC MAX phase in a facile way and exploring its potential application as electrode materials for Li-ion batteries is with great significance. In this work, we demonstrate that the V_2_SnC MAX phase particles can be prepared by the molten salt method. The prepared V_2_SnC MAX phase with uniform and refined particle size exhibit high lithium storage capacity with high-rate and excellent cyclability performance as lithium storage anode.

## Experimental

### Raw Materials

Elemental powders of vanadium (~ 300 mesh, 99.5 wt% purity), tin (~ 300 mesh, 99.5 wt% purity), and graphite (~ 300 mesh, 99.5 wt% purity) were commercially obtained from Target Research Center of General Research Institute for Nonferrous Metals, Beijing, China. Sodium chloride (NaCl, 98 wt%), potassium chloride (KCl, 98 wt%), hydrochloric acid (HCl, 36.5 wt%), and absolute ethanol (C_2_H_6_O, 98 wt%) were commercially obtained from Aladdin Chemical Reagent, China.

### Preparation of V_2_SnC

The powders were mixed in a stoichiometric ratio of V:Sn:C = 2:1.1:1 (The melting point of Sn was relatively low, we increased the content of tin because of the weight loss of tin at a high-temperature, as in the preparation of V_2_(Sn,A)C MAX phases) [20]. The starting powders of V, Sn, and graphite are mixed with inorganic salt (NaCl + KCl). After grounding for 10 min, mixture powders were put into an aluminum oxide boat, and then the alumina boat was put into a tube furnace and heated to 1000 °C during 3 h with a heating rate of 10 °C min^−1^ under an argon atmosphere, respectively. After the end of the reaction, the product is washed, filtered, and dried at 40 °C in vacuum.

### Characterizations and Measurements

The phase composition of the samples was analyzed by X-ray diffraction (XRD, D8 Advance, Bruker AXS, Germany) with Cu Kα radiation. X-ray diffractograms were collected at a step size of 0.02° 2θ with a collection time of 1 s per step. The microstructure and chemical composition were observed by scanning electron microscopy (SEM, QUANTA 250 FEG, FEI, USA) equipped with an energy-dispersive spectrometer (EDS), and the EDS values were fitted by XPP (extended Puchou/Pichoir). Structural and chemical analysis was carried out by high-resolution high-angle annular dark-field scanning transmission electron microscopy (HAADF-STEM), and lattice resolved energy-dispersive X-ray spectroscopy (EDS) using the Linköping double corrected FEI G2 Titan^3^ 60–300 microscope operated at 300 kV, and STEM-EDS was recorded with the embedded high sensitivity Super-X EDX detector. The Rietveld refinement of powder XRD pattern of V_2_SnC was by Total Pattern Solution (TOPAS-Academic V6) software.

### Electrochemical Tests

The electrochemical tests were carried out on CR2032 coin-type cells. The MAX phase electrodes were prepared by mixing 90 wt% active materials, 5 wt% acetylene black, and 5 wt% PVDF in N-methylpyrrolidinone (NMP), and the slurry mixture was then coated on Cu foil with a mass loading of 1.13 mg cm^−2^. After coating, the electrodes were dried at 60 °C for 30 min to remove the solvent. The electrodes were then cut into disks with a diameter of 12 mm, and vacuum dried at 100 °C for 12 h and weighed before assembly. All cells were assembled in Argon atmosphere glovebox with oxygen and water content less than 0.01 ppm. The lithium metal foil was used as counter and reference electrode and 1 M Li–PF6 in EC: DMC (volume 1:1) solvent as the electrolyte, glass fiber membrane as the separator. Cyclic voltammetry and impedance spectroscopy tests were carried out using a Metrohm AutoLab M204 electrochemical workstation, and the charge–discharge experiments were performed on a Neware battery test system.

### Theoretical Calculations

The theoretical calculations are performed in the framework of density functional theory with projector augmented wave method by implementing the Vienna Ab-initio Simulation Package (VASP). The generalized gradient approximation (GGA) proposed by Perdew, Burke and Ernzerhof (PBE) is selected as the exchange–correlation potential with the DFT-D3 van der Waals correction. Brillouin zone integrations are performed on 3 × 3 × 1 k-meshes for geometry optimization and electronic structure calculations. The cutoff energy of plane wave basis is set to 500 eV, and the Kohn–Sham equations is set to 10^–5^ eV which make the results reliable. To simulate the real surface circumstance, a 2 × 2 supercell of MAX phases with six layers is built to investigate the adsorption of Li and vacuum layer of 2–nm-thickness is found to be sufficient to avoid unphysical interaction between images due to the periodic boundary conditions.

## Results and Discussion

### Characterization of V_2_SnC MAX Phase

**Fig. 1:**
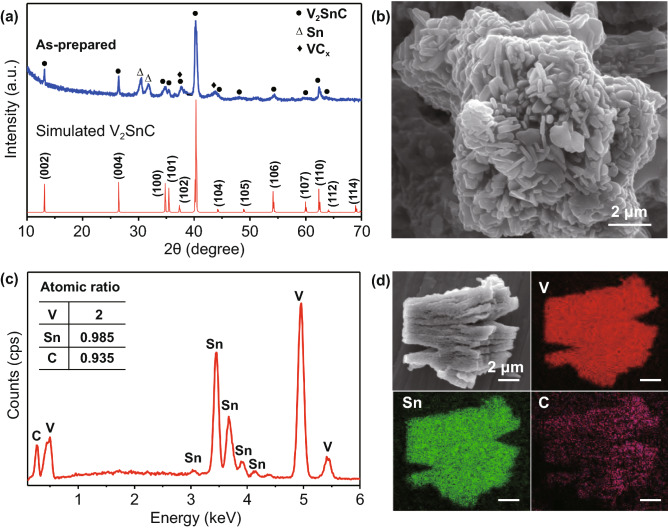
**a** The experimental measured and simulated X-ray diffraction (XRD) patterns of V_2_SnC MAX phase. **b** SEM image and **c** the corresponding energy-dispersive spectroscopy (EDS) spectrum of V_2_SnC. **d** Elemental mapping clearly proved the uniform distribution of V, Sn, and C element

The XRD patterns were shown in Fig. 1a (blue line). The characteristic peaks typical of M_2_AX phases located at 2θ ≈ 13°, 2θ ≈ 26°, and 2θ ≈ 41° indicate the synthesis of V_2_SnC is synthesized. In addition, small mass fraction of Sn metal and VC_*x*_ can be identified. In comparison with experimental result, the simulated XRD pattern of V_2_SnC (red line) coincides well with the experimentally measured pattern, which further supports the phase identification of V_2_SnC MAX by molten salt method. Moreover, the Rietveld refinement of the XRD pattern of V_2_SnC is shown in Fig. S1, the reliability factor is *R*_wp_ = 9.54% indicates the good agreement between fitting results and measured data. A small amount (2.39 wt%) of Sn metal was determined. The lattice parameters of the V_2_SnC are *a* = 2.98 Å and *c* = 13.46 Å. The atomic positions of the V_2_SnC determined form the Rietveld refinement are listed in Table S1.

Figure 1b is an SEM image of the obtained V_2_SnC powders. The V_2_SnC particles are plate-like, with a smooth surface that is similar to Ti_3_SiC_2_ synthesized by molten salt method [6]. The powder particles with length and thickness less than 0.2 µm are agglomerated in clusters of about 10 µm. As previously reported [12], the submicrometer size of the prepared MAX phase thin flakes may benefit the electrochemical performance as the LIB anode. EDS analysis (Fig. 1c) confirms the presence of V, Sn, and C elements, and the relative atomic composition of V_2_Sn_0.98_C_0.93_, that is, very close to the composition of M_2_AC (211) type MAX phases. In addition, the energy-dispersive X-ray spectrometry (EDS) elemental mapping for V_2_SnC in Fig. 1d indicates a homogeneous distribution of V, Sn, and C elements.

**Fig. 2:**
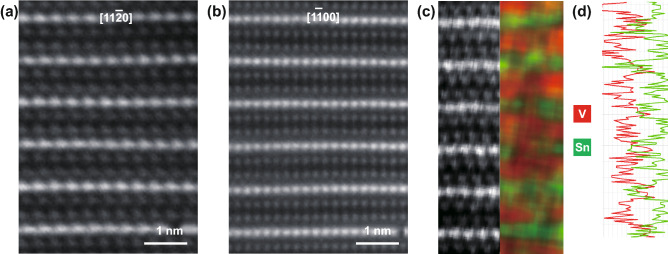
High-resolution (HR)-STEM images of V_2_SnC showing atomic positions along **a** [11 $\stackrel{\mathrm{-}}{2}$ 0] and **b** [1 $\stackrel{\mathrm{-}}{1}$ 00] direction, respectively. **c** STEM-EDS mapping of V–*K*_α_ (red) and Sn-*K*_α_ (green) signals, respectively, in [11 $\stackrel{\mathrm{-}}{2}$ 0] projection. **d** EDS line-scan extracted from the data in **c**

To further determine the crystal structure and elemental sites of the V_2_SnC MAX phase, the material was further identified by high-resolution high-angle annular dark-field scanning transmission electron microscopy (HAADF-STEM) and lattice resolved energy-dispersive X-ray spectroscopy (EDS). STEM images of the V_2_SnC phase along the [11 $\stackrel{\mathrm{-}}{2}$ 0] and [1 $\stackrel{\mathrm{-}}{1}$ 00] directions are shown in Fig. 2a, b, respectively. Along the vertical direction of both images, it can be observed that single layers of atomic columns (the A layers) are interleaved by two adjacent layers of dark atomic columns (the M layers). Carbon is typically not visible because of its weak electron scattering nature as compared to the heavier M and A atoms [21]. Except for the different relative brightness between elements, owing to mass-enhanced contrast, the structure is equivalent to the configuration of other M_2_AX phases and exhibit the characteristic zig-zag stacking of M_*n*+1_X_*n*_ slabs along [11 $\stackrel{\mathrm{-}}{2}$ 0] zone axes, which was also observed for other MAX phases [22–24]. Furthermore, the STEM-EDS elemetal maps analysis in Fig. 2c indicates only V (red) and Sn (green) in the prepared MAX phase (C is not feasible to map out), suggesting that the M and A sites consist exclusively of V and Sn, respectively, which is consistent with XRD results, indicates that the 211 type V_2_SnC MAX phase is synthesized. Figure 2d further confirms this observation by integrating the elemental map in Fig. 2c to a line-scan of the V – *K*_α_ and Sn – *K*_α_ EDS peaks. Therefore, we have established the successful preparation of the V_2_SnC MAX phase by the molten salt method.

### Electrochemical Performance of V_2_SnC MAX Phase

**Fig. 3:**
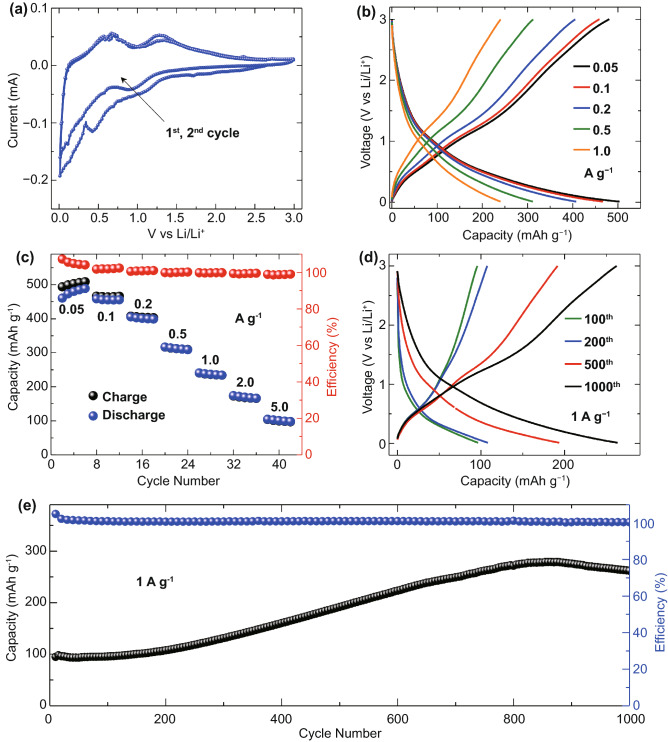
Electrochemical characterization of V_2_SnC materials: **a** Cyclic voltammetric profiles at the 1st and 2nd cycle at 0.1 mV s^–1^ within potential from 0.01 to 3 V vs. Li/Li^+^. **b** Galvano charge–discharge profiles recorded at current densities range from 0.05 A g^−1^ to 1 A g^−1^. **c** Capacities and coulombic efficiency at various current densities of the electrode. **d** Galvano charge–discharge profiles at the 100^th^, 200th, 500th , and 1000th cycle. **e** cycling at 1 A g^−1^ for 1,000 cycles

To investigate the electrochemical behavior, the V_2_SnC working electrodes were prepared with addition of 5 wt% acetylene black and 5 wt% PVDF, and lithium metal foil was used counter and reference electrode. Figure 3a shows the cyclic voltammetry profiles of the V_2_SnC MAX phase electrode at a scan rate of 0.1 mV s^−1^ within potential range from 0.01 to 3 V versus Li/Li^+^. During the first cathodic process, the irreversible capacity below 1.0 V versus Li/Li^+^ is observed for the first cycle that can be explained by the irreversible contribution of the solid electrolyte interface (SEI) layer formation as previously reported [18]. Galvano charge–discharge measurements at current densities ranging from 0.05 to 5 A g^−1^ were carried out after cycling tests. As presented in Fig. 3b, c, the V_2_SnC electrode shows the feasibility to deliver a maximum reversible capacity of 490 mAh g^−1^ at 0.05 A g^−1^, corresponding to a reversible uptake of 4.25 Li^+^ per V_2_SnC unit, which is much higher than the previous reported results [18]. It should be noted that the electrochemical performance of the V_2_SnC was obtained with an electrode contains that only 5 wt% acetylene black while previous reported studies were investigated with at least 10 wt% of conducting carbon [12, 18]. That is, compared to other lithium storage anodes such as silicon or metal oxides [25–27], a lower amount of conducting carbon is needed for the metallic conducting V_2_SnC electrode. The decrease of conducting carbon may be important for increasing the volumetric capacity considering the low density of carbon as compare to MAX phases. A maximum volumetric capacity of 570 mAh cm^−3^ was achieved with a 10-um-thick electrode. In addition, the capacity retains 100 mAh g^−1^ at 5 A g^−1^, indicating the high-power capability of the electrode. The high capacity and high-power could be ascribed to the small particle size (few hundreds nanometers, Fig. S3) of molten salt derived V_2_SnC MAX phase after ball milling treatment. The smaller size of the MAX phase particles enables more Sn atoms to be exposed to the electrolyte and induce alloy formation with the lithium ions. To test this hypothesis, microsize V_2_SnC particle (tens of micrometers) without ball milling was investigated for comparison. XRD patterns (Fig. S3a) of V_2_SnC before and after ball milling confirm that the samples retain the V_2_SnC phase after ball milling, but the peak intensities decreases and the beaks are broader, indicating the reduction of particle size after ball milling [28, 29], which is confirmed by SEM (Fig. S3b, c) and particle size analyzer test (Fig. S3e). Figure S4 presents the electrochemical characterization of micro size V_2_SnC particle. Cyclic voltametric profiles are the same as the nano size V_2_SnC particle after ball milling as presented in Fig. 3a, however, a maximum capacity of 210 mAh g^−1^ (Fig. S4c) is much lower than the electrode with nano size V_2_SnC particle, which highlights the importance of decreasing particle size to achieve high electrochemical performance.

The Galvano charge–discharge (GCD) cycling profiles in Fig. 3d with a current density of 1 A g^−1^ demonstrate the increase of the capacity from 100 mAh g^−1^ at the 100th cycle to 260 mAh g^−1^ at 1000th cycle. The deceasing size of MAX phases particles that causes by Sn–Li (de)alloying reaction during (de)lithiation as described by Zhao et al*.* [18] is believed to be responsible for the increase of the capacity during cycling. The maximum capacity of 280 mAh g^−1^ was achieved at the 800th cycle, however, further cycling tests witness a slightly capacity decrease that may cause by the abscission of Sn atom at the particle edge after repeating (de)alloying reaction.

### Charge Storage Mechanism of V_2_SnC Electrode

**Fig. 4:**
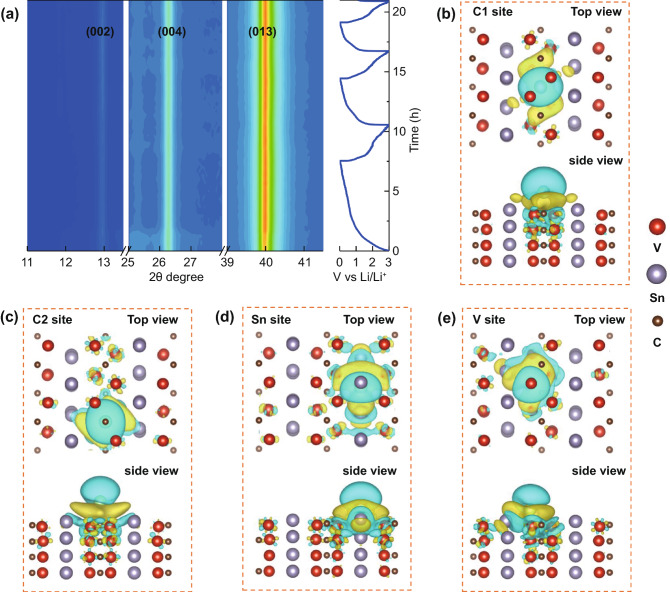
**a** *Openrando* XRD patterns of V_2_SnC electrode during the first three cycles. Charge redistributions due to the interaction with Li on **b** C_1_ (out-plane C), **c** C_2_ (second-plane C), **d** Sn, and **e** V adsorption sites. Yellow/blue color represents the charge accumulation/depletion, where the isosurfaces refer to an isovalue of 6 × 10^–3^ eletrons/bohr^3^

To further confirm our speculations on the charge storage mechanism, *operando* and *ex-situ* XRD investigations of V_2_SnC electrode during charge–discharge process were performed. As shown in Figs. 4a and S5, the XRD diffraction peaks of V_2_SnC MAX phase do not show noticeable change during several charge–discharge cycles, evidencing the stable crystal structure of V_2_SnC MAX phase without phase transition or evolution of lattice constant [30]. This is consistent with the previous study of Nb_2_SnC explained by the Li-Sn alloying only at the edges of Nb_2_SnC particles, whereas Sn in the bulk was not involved in the redox process [18]. The residual Sn metal may contribute to the Li^+^ storage capacity, however, the low content of 2.39 wt% results in limited Li^+^ storage capacity contribution [16]. Figures S6–S8 present SEM, TEM images and EDS mapping results the V_2_SnC particles after electrochemical cycling tests. V, Sn, C elements are well and evenly distributed on the particle. The high-resolution TEM (HRTEM) image shows clear lattice fringe spacing of 0.67 nm (Fig. S8d), corresponding to the (002) lattice plane of V_2_SnC, further confirming the stable structure of V_2_SnC and agreeing well with the proposed charge storage mechanism, indicating the outstanding stability of V_2_SnC electrode during cycling [31]. Such a charge storage mechanism is quite different from the typical charge storage mechanism of battery electrodes with phase transition or lattice constant change. It is believed that the stable crystal structure without phase transition or lattice constant evolution is the origin of the cycling stability and high-rate performance of V_2_SnC electrode. The edge Sn alloy reaction mechanism explains that the lithium storage capacity of Sn-based MAX phase could be further increase by reducing the particle size and exposing more Sn atom to the electrolyte.

To gain insight into the charge storage mechanism from the atomic scale, DFT calculations were performed. In order to evaluate the lithium storage mechanism of V_2_SnC, the preferred adsorption sites of Li ions have been calculated on the 2 × 2 × 1 supercell surface, as shown in Fig. 4b–e. The adsorption energies *E*_ad_ on the top sites of C_1_ (out-plane C), C_2_ (second-plane C), Sn, and V are −0.754, −0.38, −0.448, and −1.255 eV, respectively. Noticeable, the Li adsorbed only in second-plane Sn and V indicating the potential energy surface is in line with the geometric construction. It is the special zig-zag edge structure that makes the adsorptions more preferable. To investigate the limitation of adsorptions, we locate the Li ions on the top of such sites, and the results show that the first-plane V will trap all the Li on the top of C due to the strong interaction between the Li and V. Besides, the Sn prefer to protruding out of surface which makes energic favorable adsorption.

To gain a deeper insight into the diverse adsorption behaviors, Bader charge and charge redistribution were performed. Compared to the inside of V_2_SnC, the change of Bader charge in surface elements shows the interaction between the host materials and guest cation. V_2_C layer of V_2_SnC gains charge ranges from 0.022 to 0.029 |*e*| with one Li cation adsorbed on V_2_C layer (C_1_, C_2_, V sites). On the other hand, Sn gains 0.026 |*e*| when one Li cation adsorbed on Sn adsorption site. These results indicate that the V_2_C-Li and Sn-Li reactions are both preferable to occur and contribute to the redox capacity. The top and side views of charge redistribution from C_1_, C_2_, Sn, and V at the same isosurface value (6 × 10^–3^ electrons bohr^−3^) are shown in Fig. 4c–e. The yellow/blue color represents the charge accumulation/depletion, and the electron cloud represents the degree of interaction. It is obvious that a strong interaction between the Li and Sn, V, suggesting the dual redox reaction of V_2_C–Li and Sn–Li as speculated in the previous discussion (Table 1).

**Table 1:** Bader charge of V_2_SnC with one Li adsorbed at different sites

Adsorption sites	V–C layer (△)	Sn layer (△)	Li
unadsorption	−0.454 (0)	0.455 (0)	/
C_1_	−0.425 (0.029)	0.462 (0.007)	−0.858
C_2_	−0.428 (0.026)	0.463 (0.008)	−0.842
V	−0.432 (0.022)	0.466 (0.011)	−0.829
Sn	−0.446 (0.008)	0.481 (0.026)	−0.844

The positive/negative values represent positively/negatively charged. The values of △ represent the Bader charge gain or depletion

## Conclusions

V_2_SnC was prepared by the molten salt method. A maximum lithium storage capacity up to 490 mAh g^−1^ (570 mAh cm^−3^) was achieved with the V_2_SnC MAX phase electrode, surpassing the highest capacities reported of the MAX phase anodes. The superior capacity is achieved from the reduced size of molten salt derived V_2_SnC particles, while the high-rate and good cyclability is because of the stable crystal structure of V_2_SnC MAX phase during cycling. A charge storage mechanism with dual redox core reaction renders these laminated MAX phases with lithium alloying elements (Si, Sn, S, and so on) very interesting to be explored as high-performance lithium storage anodes.

## Supplementary Information

Below is the link to the electronic supplementary material.

Supplementary file1 (PDF 750 KB)
