# Supplementary material for: Electrochemical Lithium Storage Performance of Molten Salt Derived V2SnC MAX Phase
Source: Nanomicro Lett. 2021 Jul 22;13:158. doi: 10.1007/s40820-021-00684-6 (PMC8298715; doi:10.1007/s40820-021-00684-6)
Supplement: Supplementary file 1 — Supplementary file1 (PDF 750 KB) [file 40820_2021_684_MOESM1_ESM.pdf]

Supporting Information for

Electrochemical Lithium Storage Performance of Molten Salt Derived V_2SnC MAX Phase

Youbing Li[1, 2, #], Guoliang Ma[3, #], Hui Shao[4], Peng Xiao[3], Jun Lu[5], Jin Xu[6], Jinrong Hou[7], Ke Chen[1, 2], Xiao Zhang[1, 2], Mian Li[1, 2], Per O. Å. Persson[5], Lars Hultman[5], Per Eklund[5], Shiyu Du[1, 2], Zhifang Chai[1, 2], Zhengren Huang[1, 2], Na Jin[3], Jiwei Ma[7], Ying Liu[3], Zifeng Lin[3, *], Qing Huang[1, 2, *]

[1]Engineering Laboratory of Advanced Energy Materials, Ningbo Institute of Industrial Technology, Chinese Academy of Sciences, Ningbo, Zhejiang 315201, P. R. China

[2]Qianwan Institute of CNiTECH, Zhongchuangyi Road, Hangzhou bay District, Ningbo, Zhejiang, 315336, P. R. China

[3]College of Materials Science and Engineering, Sichuan University, Chengdu, 610065, P. R. China

[4]CIRIMAT UMR CNRS 5085, Université Toulouse III- Paul Sabatier, 118 route de Narbonne, 31062 Toulouse Cedex 9, France

[5]Department of Physics, Chemistry, and Biology (IFM), Linköping University, Linköping 58183, Sweden

[6]School of Machine Engineering, Dongguan University of Technology, Dongguan, 523808, P. R. China

[7]Institute of New Energy for Vehicles, School of Materials Science and Engineering, Tongji University, Shanghai 201804, P. R. China

[#]Y. B. Li and G. L. Ma contributed equally to this work

*Corresponding authors. E-mail: linzifeng@scu.edu.cn (Zifeng Lin);

huangqing@nimte.ac.cn (Qing Huang)

Supplementary Figures and Tables

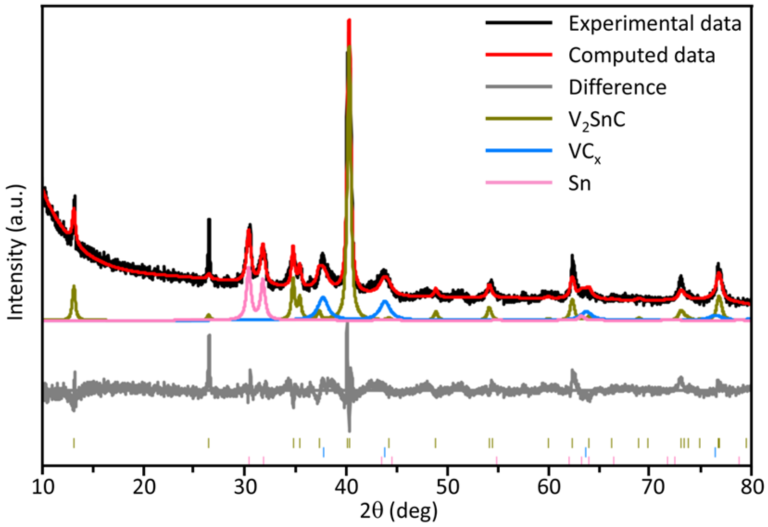

Fig. S1 Comparison between the experimental (black crosses) and calculated XRD (red line) patterns of V_2SnC. The deviation plot (gray line) is shown in the lower part of Fig. 1a. The brown, pink, and blue ticks below the pattern represent the peak positions of the V_2SnC, Sn, and VC_x phases, respectively.

Table S1 Atomic positions in V_2SnC determined from the Rietveld refinement

Site	Element	*x*	*y*	*z*	Symmetry	Wyckoff symbol
M1	V	1/3	2/3	0.0881	3m	4f
Al	Sn	1/3	2/3	0.75	-6m2	2d
C1	C	0	0	0	-3m	2a

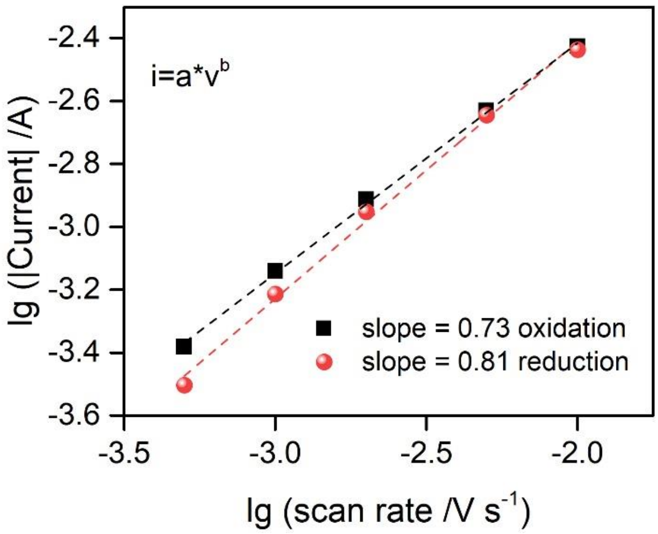

Fig. S2 *b* values calculated from CVs at oxidation peaks (~0.6 V) and reduction peaks (~1.1 V). *b* value of cathodic process is 0.81 and anodic process is 0.73, suggesting the combination of diffusion-control and non-diffusion-control behavior of the V_2SnC electrode.

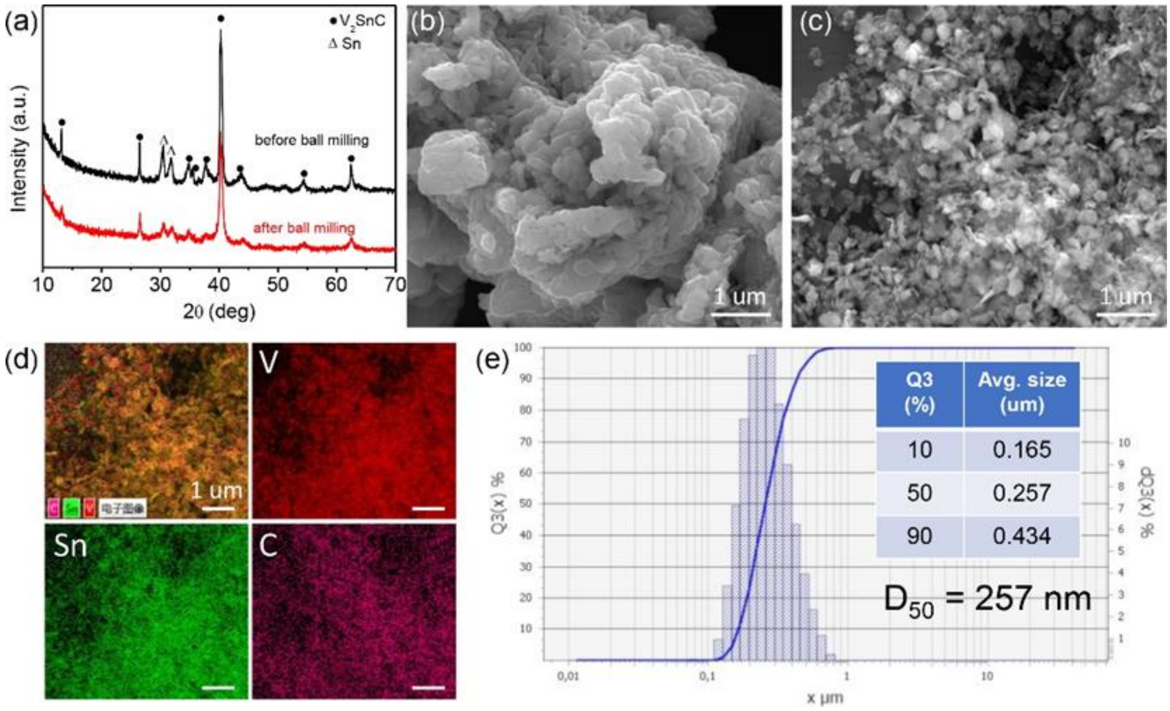

Fig. S3 a XRD patterns of V_2SnC MAX phase before and after ball milling. SEM image of V_2SnC **b** before and **c** after ball milling. **d** Elemental mapping clearly proved the uniform distribution of V, Sn, and C element. **e** Particle size distribution of V_2SnC after ball milling.

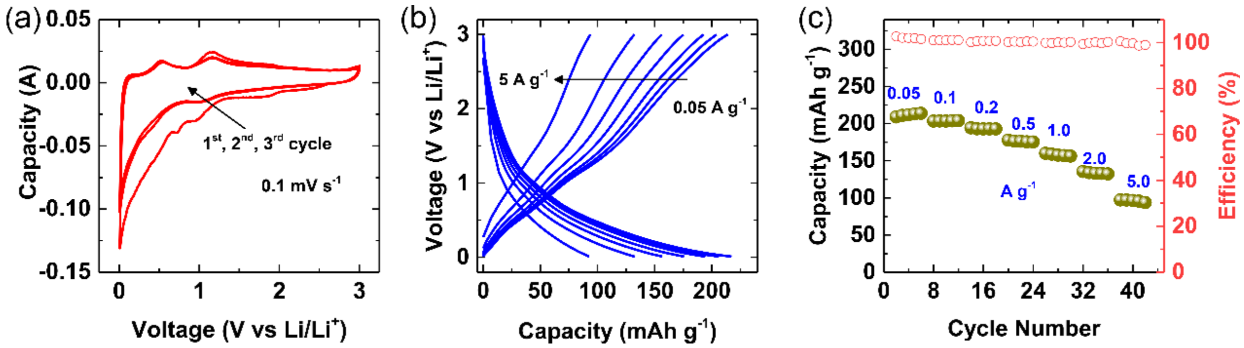

Fig. S4 Electrochemical characterization of V_2SnC materials without ball milling: **a** Cyclic voltametric profiles at the 1st, and 2nd cycle at 0.1 mV s^{-1} within potential from 0.01 to 3V vs. Li/Li^{+}. **b** Galvano charge-discharge profiles recorded at current densities range from 0.05 to 5 A g^{-1}. **c** Capacities and coulombic efficiency at various current densities of the electrode

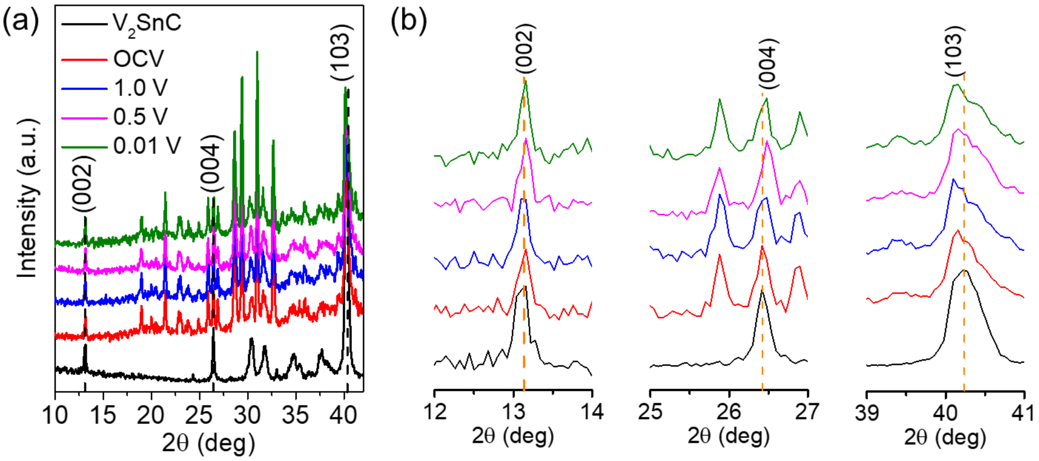

Fig. S5 a *ex-situ* XRD patterns and **b** magnified XRD patterns at (002), (004) and (103) diffraction peaks of V_2SnC electrode at different potentials

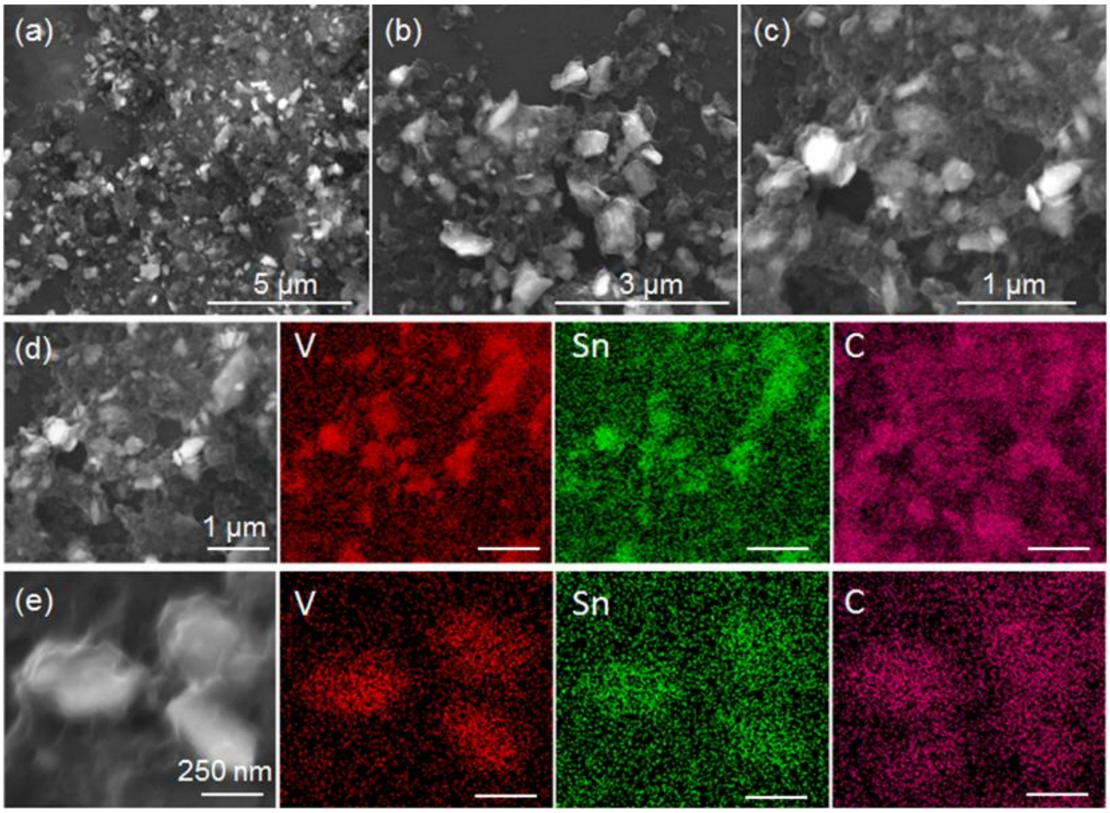

Fig. S6 SEM results of V_2SnC electrode after long cycle electrochemical tests. **a-c** SEM image of V_2SnC at different magnifications. **d-e** Elemental mapping clearly proved the uniform distribution of V, Sn, and C element.

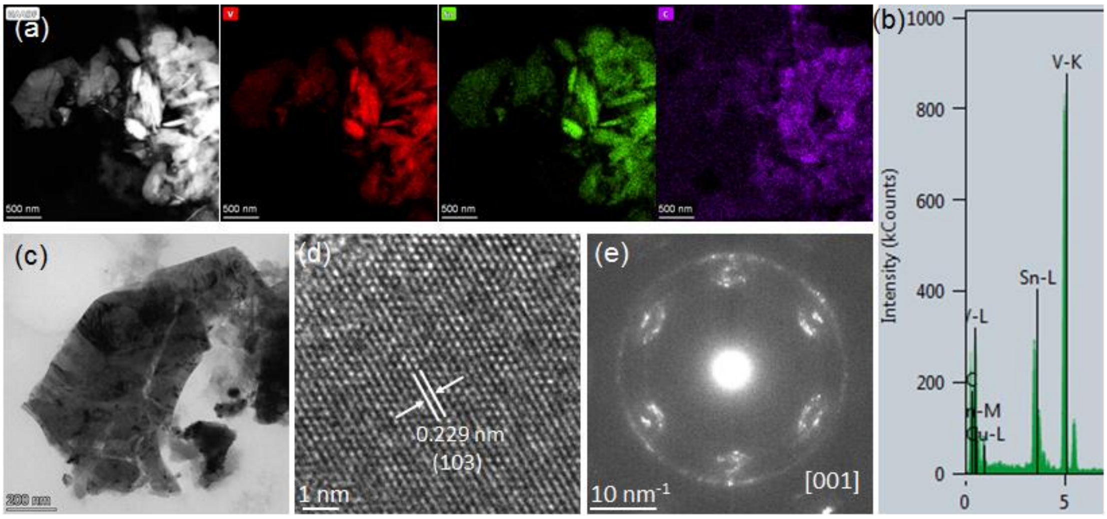

Fig. S7 TEM results of V_2SnC electrode after long cycle electrochemical tests. **a** Elemental mapping of V-Kα, Sn-Kα and C-Kα elements of V_2SnC. **b** TEM-EDS of the V_2SnC. **c–d** HR-TEM image and **e** Selected area electron diffraction pattern of V_2SnC

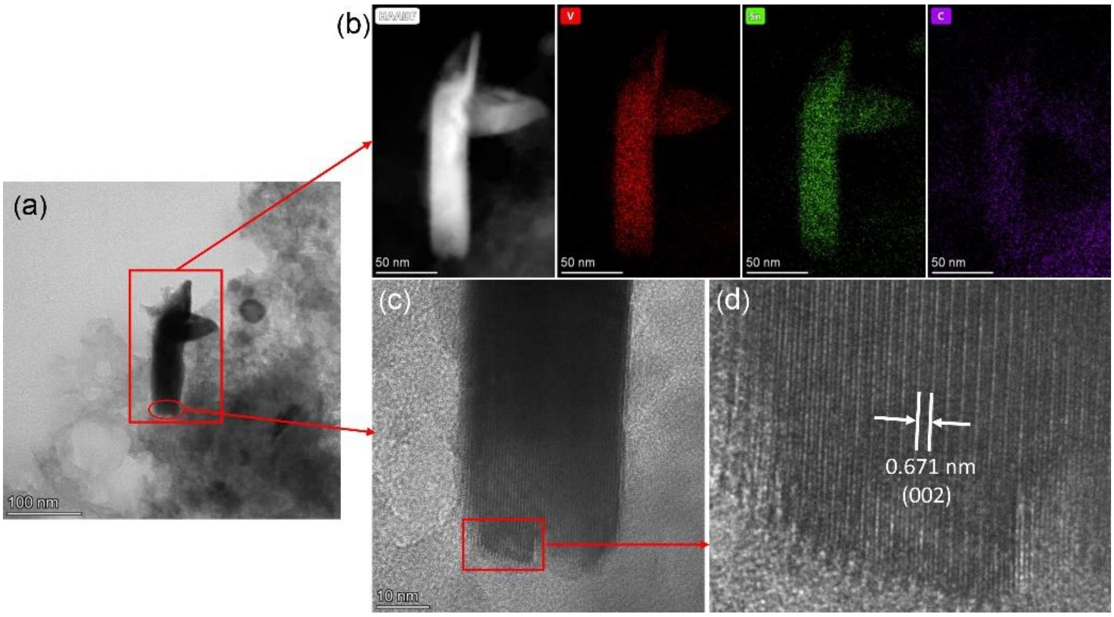

Fig. S8 TEM results of V_2SnC electrode after long cycle electrochemical tests. **a** TEM image and **b** corresponding element mapping distribution. **c** HR-TEM image and **d** magnified TEM image of V_2SnC
